# Assessment of dedicated MRI sequences in the evaluation of the acromioclavicular joint dislocation—A retrospective study

**DOI:** 10.1007/s00256-026-05176-5

**Published:** 2026-03-23

**Authors:** Antoine Bagnoud, Philippe Vial, Carlo Melis, Harriet Thoeny, Julien Galley

**Affiliations:** 1 Department of Radiology, Faculty of Science and Medicine of the University of Fribourg, Hôpital Cantonal Fribourgeois (HFR), Chemin Des Pensionnats 2/6, 1752 Villars-Sur-Glâne, Switzerland; 2 Department of Orthopedic Surgery, Hôpital Cantonal Fribourgeois (HFR), Chemin Des Pensionnats 2/6, 1752 Villars-Sur-Glâne, Switzerland; 3 Department of Radiology, Hôpital Cantonal Fribourgeois (HFR), Chemin Des Pensionnats 2/6, 1752 Villars-Sur-Glâne, Switzerland

**Keywords:** Acromioclavicular joint dislocation, ACJ, Acromioclavicular ligaments, Magnetic resonance, MRI

## Abstract

**Objectives:**

To assess the added value of two additional dedicated MRI sequences in the evaluation of the acromioclavicular ligament complex in patients with traumatic acromioclavicular joint injury.

**Materials and methods:**

In this single-center study, shoulder MRIs of post-traumatic patients with suspected acromioclavicular joint dislocation were retrospectively evaluated twice by four readers of varying levels of expertise: once with the standard protocol and once with two additional dedicated acromioclavicular ligament sequences (a proton density-weighted sequence parallel to the acromioclavicular joint and a coronal oblique PD-weighted sequence, with a slice thickness of 2 mm). The acromioclavicular and the coracoclavicular ligaments, along with their different bundles, were analyzed. Intrareader reliability and interreader agreement were assessed by intraclass correlations. Associated shoulder lesions were reported.

**Results:**

A total of 85 shoulder MRIs from patients with acute trauma were retrospectively analyzed (mean age 38.4 ± 13.6 years, male = 73 [85.9%]). Acromioclavicular dislocations were reported in 75 patients (88.2%). The intrareader reliability was good to excellent, ranging from 0.81 to 0.90. Overall, the interreader agreements were excellent (intraclass correlations = 0.93 and 0.94 at both four and six sequences, respectively). The less experienced reader agreed with the more experienced one with and without dedicated acromioclavicular sequences (significant equivalence: *t*[12] = 2.946, *p* = 0.006).

**Conclusion:**

In this retrospective cohort, adding dedicated acromioclavicular joint sequences did not show evidence of improvement in the detection and the characterization of potential acromioclavicular ligament lesions, regardless of the reader’s level of experience.

**Supplementary Information:**

The online version contains supplementary material available at https://doi.org/10.1007/s00256-026-05176-5.

## Introduction

Acromioclavicular joint (ACJ) dislocation appears to be common, representing about 10% of all shoulder injuries [1, 2]. Since 1984, the X-ray Rockwood classification is used for the ACJ dislocation, based on the direction and the degree of osseous displacement seen on conventional radiograph. Currently, most acromioclavicular injuries are managed based on clinical and radiographic findings. MRI is not part of the official classification yet. However, its role is increasing, especially in the acute post-traumatic phase, by more accurately assessing the regional ligamentous structures. Indeed, some studies have shown that MRI findings may alter the Rockwood classification in a considerable number of patients, and those findings can influence therapeutic decisions [3, 4]. MRI appears to be increasingly decisive in ligament assessment, but its exact role remains to be determined.

To our knowledge, the first study to report the use of dedicated acquisition planes for examining the ligamentous structures of the ACJ, conducted by Schaefer et al. in 2005 [4], demonstrated that a coronal oblique fat-saturated proton-density (PD)-weighted plane parallel to the distal clavicle and a coronal plane orthogonal to the ACJ axis, accurately depicts the normal anatomy of the ACJ as well as the trapezoid and conoid ligaments. A RadioGraphics article published in 2008 by Alyas et al. [5] also described the coronal oblique plane obtained parallel to a line drawn between the coracoid process and the lesser humeral tuberosity. The same coronal oblique plane was later used by Nemec et al. in 2011 [3], who demonstrated that MRI findings can modify the Rockwood classification in a considerable number of patients. White et al. [6] also described the frequency and location of injuries to the superior and inferior portions of the acromioclavicular ligament in hockey players in 2020 using coronal planes. However, additional ACJ planes are not always performed in suspected ACJ injuries across different institutions, and their usefulness has never been clearly demonstrated in any study.

The aim of this study is to assess the added value of two additional dedicated MRI sequences in the evaluation of the acromioclavicular ligament complex in patients with traumatic acromioclavicular joint injury.

## Materials and methods

### Study design

The present retrospective study has been approved by the local ethics committee (CER-VD BASEC-ID 2024-00840) on May 28, 2024. This study is a monocentric, comparative study. Four readers retrospectively analyzed 85 shoulder MRIs performed between July 2, 2017, and April 12, 2024. All exams were acquired and stored in the hospital database (PACS). All data were collected and stored in REDCap (Research Electronic Data Capture).

### Patient selection

Patients between 18 and 75 years old with suspicion of acute unilateral ACJ luxation who were referred by the hospital orthopaedic department for an MRI of the shoulder were included. The protocol with dedicated ACJ sequences was selected based on clinical indications and suspicions. Patients were retrospectively included based on their MRI protocol and medical history. MRI examinations performed during the acute phase (0–21 days post-trauma) were enrolled. Only patients who had signed the hospital’s general research consent were considered. Exclusion criteria were as follows: history of prior fracture or surgery on the affected side (humerus, distal clavicle, or ACJ), atraumatic chronic pain, a medical history of rheumatoid disease or connective tissue disorders, and incomplete or inadequate imaging protocols. Population characteristics and technical parameters such as gender, age, and affected side were recorded including the time delay between trauma and MRI, as well as the MRI field strength.

### Imaging techniques and acquisition planes

**Fig. 1:**
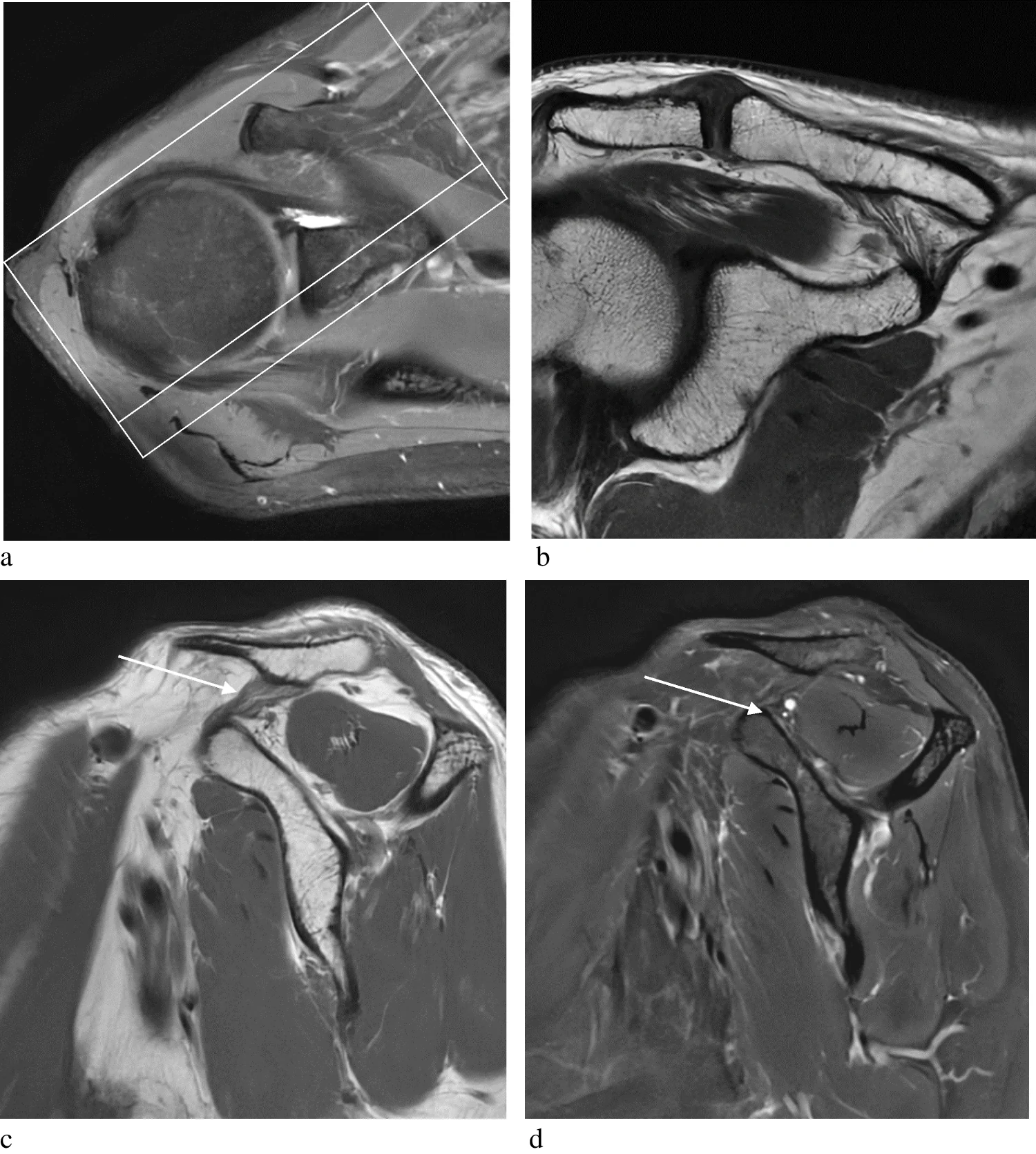
A 49-year-old woman with a normal right shoulder MRI: **a** Axial PD-weighted image showing how the additional coronal oblique sequence with a slice thickness of 2 mm is obtained: parallel to a line drawn from the coracoid process to the lesser tuberosity. **b** Coronal oblique PD-weighted sequence highlighting the coracoclavicular ligaments (arrow: trapezoid and arrowhead: conoid). **c** For comparison, sagittal T1-weighted sequence also displays coracoclavicular ligaments on a few slices (arrow). **d** Same plane as **c** in a PD-weighted sequence, not accurate for directly assessing coracoclavicular ligament fibers (arrow)

**Fig. 2:**
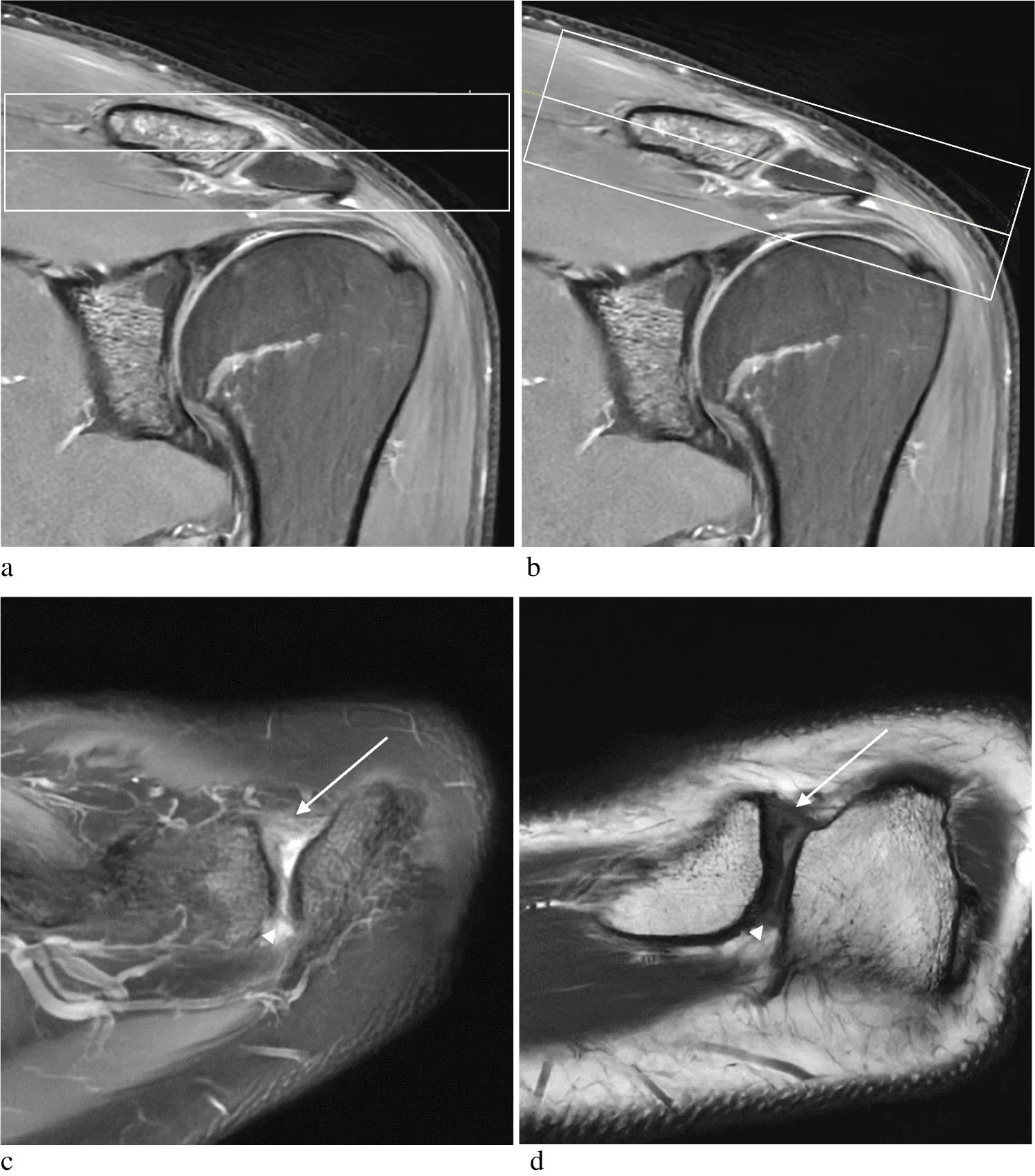
A 23-year-old woman with a normal right shoulder MRI: **a** Coronal PD-weighted image showing the standard axial acquisition plane. **b** Coronal PD-weighted image showing the additional acquisition plane parallel to the acromioclavicular joint. **c** Standard axial PD-weighted image showing the acromioclavicular ligament, particularly its anterior (arrow) and posterior (arrowhead) parts. **d** Dedicated PD-weighted sequence parallel to the acromioclavicular joint with a slice thickness of 2 mm, showing the acromioclavicular ligament, especially the anterior (arrow) and posterior (arrowhead) bundles

Examinations were performed on a 1.5 Tesla (Optima MR360) or a 3 Tesla (SIGNA Premier or DISCOVERY MR750) MR scanner, both from GE Healthcare, using a standard shoulder coil. The standard shoulder protocol included axial, coronal, and sagittal fat-saturated PD-weighted sequences, along with a sagittal nonfat-saturated T1-weighted sequence. The first added ACJ sequence was a nonfat-saturated PD-weighted sequence in the coronal oblique plane with a slice thickness of 2 mm obtained parallel to a line drawn between the coracoid process and the lesser humeral tuberosity, as described by Alyas et al. [5] (Fig. 1), providing optimal visualization of the CCL. The second dedicated ACJ sequence was a non-fat-saturated PD-weighted sequence obtained parallel to the ACJ joint with a slice thickness of 2 mm (Fig. 2), allowing optimal visualization of the anterior and posterior portions of the ACL. Details of these two additional sequences are listed in Supplementary Material 1.

### Imaging analysis

MR images were evaluated twice by four readers: one senior musculoskeletal radiologist (reader 1), one senior orthopaedic surgeon specialized in shoulder surgery (reader 2), one fellow in musculoskeletal radiology (reader 3), and one junior resident radiologist with 3 years of experience (reader 4). The first evaluation was conducted using the standard basic shoulder protocol (four sequences) across the entire cohort. Subsequently, the second evaluation was performed with the two additional ACJ dedicated sequences (six sequences in total), thereby forming two comparative groups. Results were compared both within each reader (with vs. without dedicated sequences) and between readers. In the absence of surgical correlation, the senior musculoskeletal radiologist was considered as an internal comparator.

### Outcomes/evaluation

Primary outcomes included the evaluation of the ACL based on its overall condition, followed by the assessment of its superior, inferior, anterior, and posterior bundles separately. The overall condition of the CCL was assessed, followed by a separate evaluation of the trapezoid and conoid bundles. The insertions of the trapezoid and the deltoid muscles’ insertions on the distal clavicle and the acromion were assessed. Ligament damage was characterized as “normal” (intact), “partially torn” (thickened, increased intrasubstance PD-weighted signal), or “completely torn” (complete ligament tear with visualization of two separated ends). Muscle injuries were graded as “intact” (uniform, no intramuscular fluid signal), “partial lesion” (increased intramuscular PD-weighted signal without fiber disorganization), and “partial or complete disinsertion” (muscular fiber disorganization, presence of muscle fiber stumps or free fluid between the bone and the muscle).

The secondary outcomes were the evaluation of associated shoulder lesions in patients with and without injuries of the ACJ ligamentous complex, as defined by the senior musculoskeletal radiologist (reader 1) using the four sequences protocol, such as rotator cuff tendon injury and long head of biceps tendon lesion, graded from “normal” (intact), “partial tendon injury” (thickened, intrasubstance PD-weighted signal, incomplete tear), and “complete tear” (complete tendon tear with visualization of two separated ends, ± tendon retraction). Fatty degeneration of the rotator cuff muscles was assessed using the Goutallier classification “grade 0–1” (normal), “grade 2” (less fat than muscle), “grade 3” (same amount of fat and muscle), and “grade 4” (more fat than muscle). Dislocation or subluxation of the long head of the biceps tendon was considered present if half or more of the tendon width was located outside the bicipital groove. CAL lesions were graded as other ligaments. Acute cartilage and labral damage were also evaluated. Distal clavicle, acromion, humeral head, and scapula appearances were evaluated as “intact” (no intraosseous PD-weighted hypersignal), “bone bruise” (intraosseous PD-weighted hypersignal without fracture line), or “fracture” (cortical discontinuity). Subacromial-subdeltoid bursitis was diagnosed when fluid collection extended laterally between the deltoid muscle and the proximal humerus, caudally to the greater tubercle, or when the fluid thickness within the bursa exceeded 1.4 mm [7].

### Statistical analysis

Statistics were computed using R (Foundation for Statistical Computing, Vienna, Austria). Intraclass correlations (ICCs) were computed using the R package “irr” [8]; differences between ICCs were computed using Pearson and Filon’s (1898) *z* for overlapping correlations based on dependent groups [9] with the R package “cocor” [10], assuming that a *z*-transformation is adapted to any coefficient with −1 to 1 bounds, tests of equivalence between ICCs were computed using the R package “TOSTER” [11]. ICCs were interpreted as follows (Koo and Li, 2016 [12]): below 0.50 = poor, between 0.50 and 0.75 = moderate, between 0.75 and 0.90 = good, and above 0.90 = excellent. As the weighted kappa only accepts two raters, the ICC is the most appropriate tool for this study. Different models of ICCs were used to assess intra- and interreader agreements (see below for details) following the flowchart detailed in Koo and Li [12]. All ICCs are reported alongside their 95% confidence intervals (CIs); see [13–15] and the packages’ manuals [8, 10, 11] for the CI computation. ICCs were reported for each lesion independently, for all lesions collectively, for ACL and CCL lesions specifically, and for the overall ACL and CCL condition. For the purpose of this study, the most relevant combination is the “ACL and CCL general condition,” given that the additional MRI sequences were optimized to assess these ligaments in detail, as they have important prognostic and therapeutic implications.

To determine whether the addition of sequences influenced lesion characterization by a given reader, intrareader reliability between four and six sequences was computed using two-way ICCs with single unit and agreement between raters for each lesion and each reader. As a measure of consistency, if the intrareader reliability (i.e., ICC) is high, then the addition of sequences had little impact, but if it is low, then the addition had an impact. Intrareader reliabilities were compared between each pair of readers to draw inference using a Fisher’s test on independent groups. If at least one *p*-value is observed below the significance threshold (set at 0.009 after Sidak correction for six comparisons, corresponding to all possible pairing of the four readers), it would provide evidence that the impact of the added sequences is influenced by the reader’s experience. A priori power analysis computed with the R package “cower” [16] showed that to observe a difference between good and excellent ICCs (0.75 vs. 0.90), 66 data points would be needed to reach 80% power with a two-sided Fisher’s test.

To assess if the addition of sequences altered interreader agreement, two-way ICCs with average unit and consistency across raters were calculated for each lesion across all four readers, with both the 4-sequences and the 6-sequences protocols. Higher ICCs indicate stronger agreement among readers. As additional sequences were hypothesized not to affect interreader agreement, equivalence between ICCs was assessed with a dependent two one-sided *t*-test (TOST) with default margins of Cohen’s *dz* = 0.5.

## Results

**Fig. 3:**
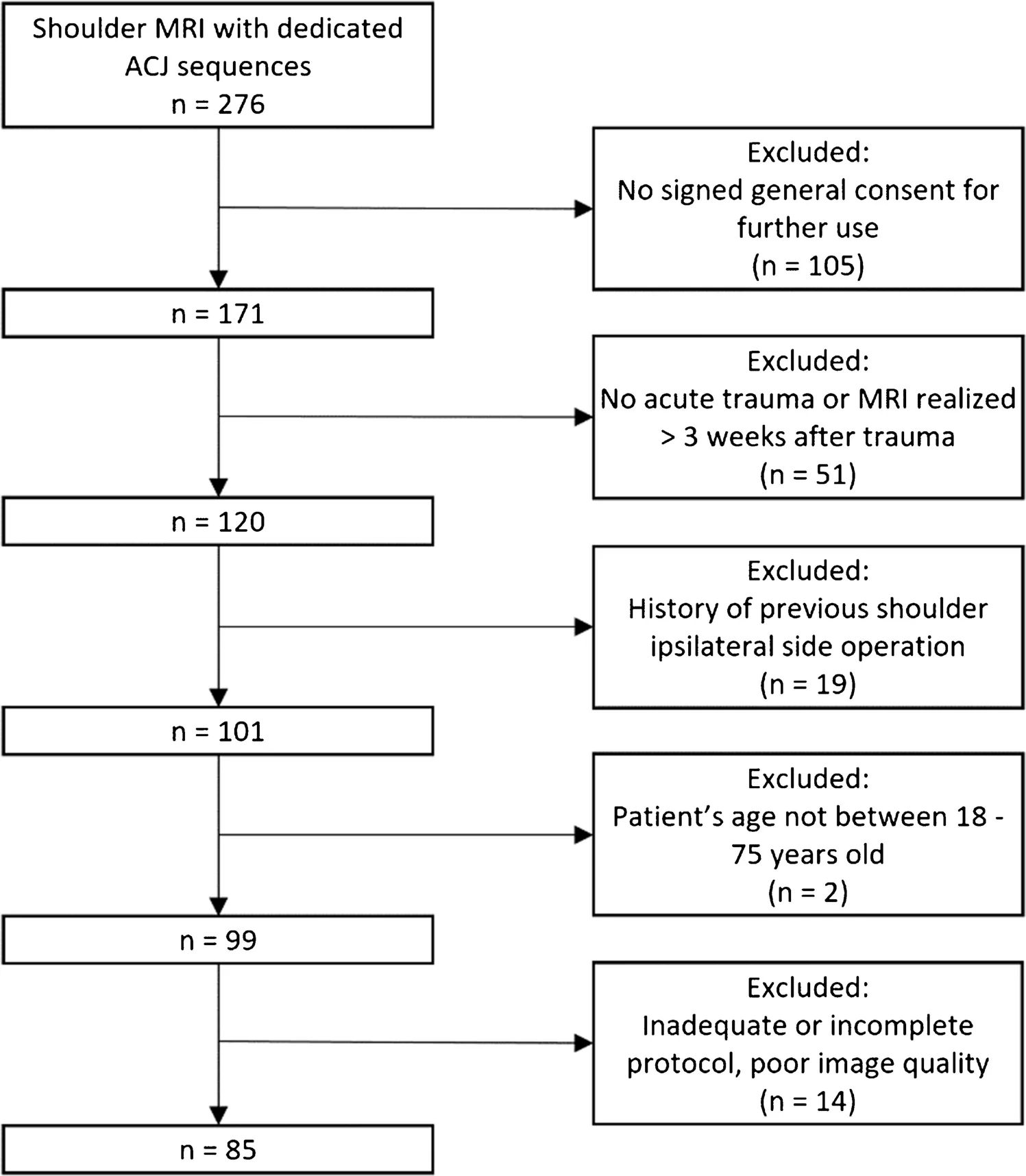
Flow chart of the study

Eighty-five post-traumatic shoulder MRIs were retrospectively evaluated between September 2024 and February 2025. Initially, 276 shoulder MRIs performed before the 7th of July 2024 (ranging from 2012 to 2024) were retrospectively identified based on the realization of dedicated ACJ sequences. Of these, 191 were ineligible according to inclusion and exclusion criteria as shown in Fig. 3.

**Fig. 4:**
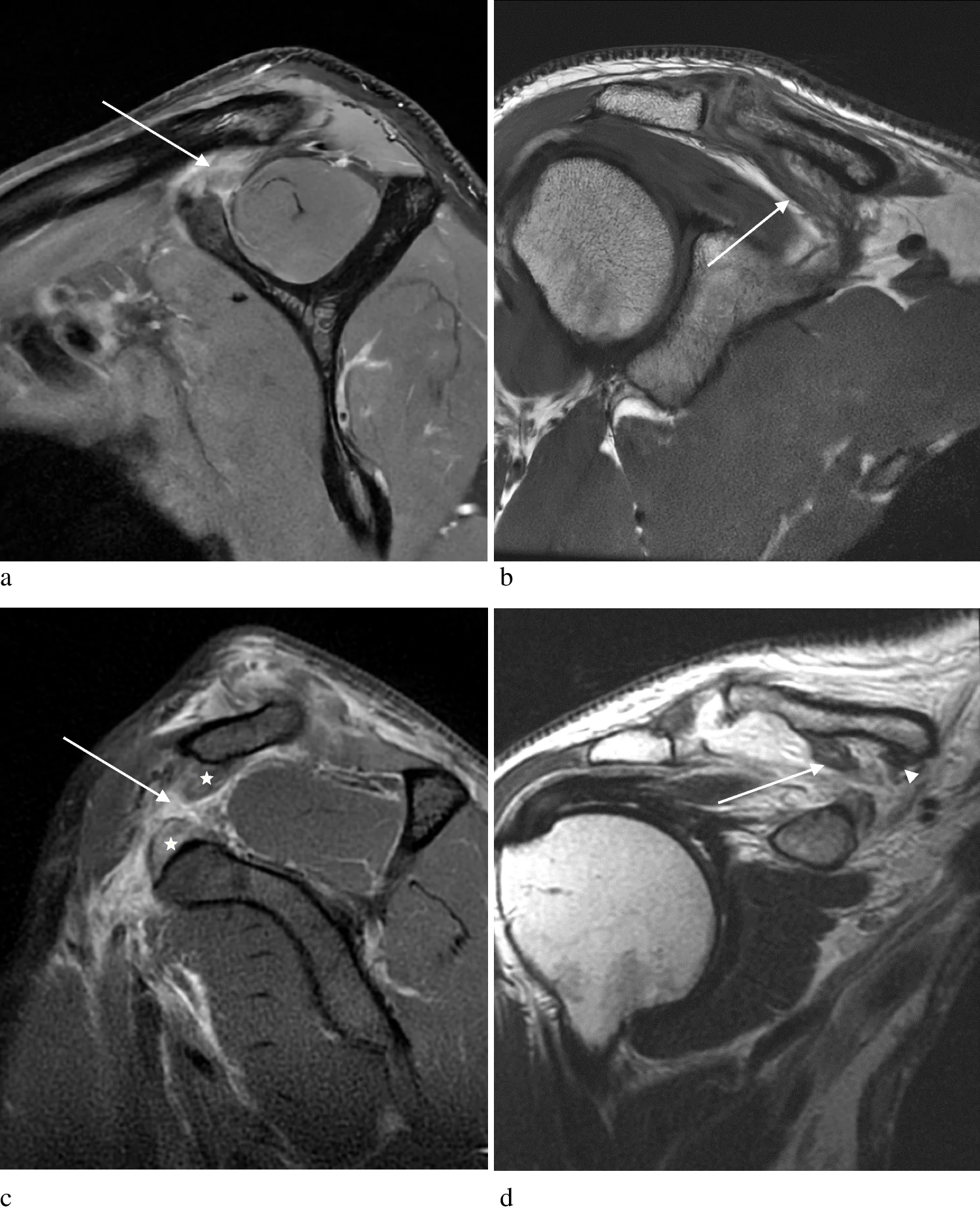
**a** A 31-year-old woman with right shoulder pain following a skiing fall. MR image of the right shoulder on a sagittal PD-weighted sequence, showing a sprained coracoclavicular ligament with edema and some persistent intact fibers (arrow). **b** The same injury on a coronal oblique PD-weighted sequence, with ligament thickening and incomplete disruption (arrow), classified as Rockwood type II. **c** A 48-year-old woman with right shoulder pain following a trauma. MR image of the right shoulder on a sagittal PD-weighted sequence, showing a completely torn coracoclavicular ligament with fluid (arrow) and two completely separated ligament ends (asterisks). **d** The same case on a coronal oblique PD-weighted sequence, showing complete disruption of the trapezoid (arrow) and the conoid ligament (arrowhead), classified as Rockwood type III

**Fig. 5:**
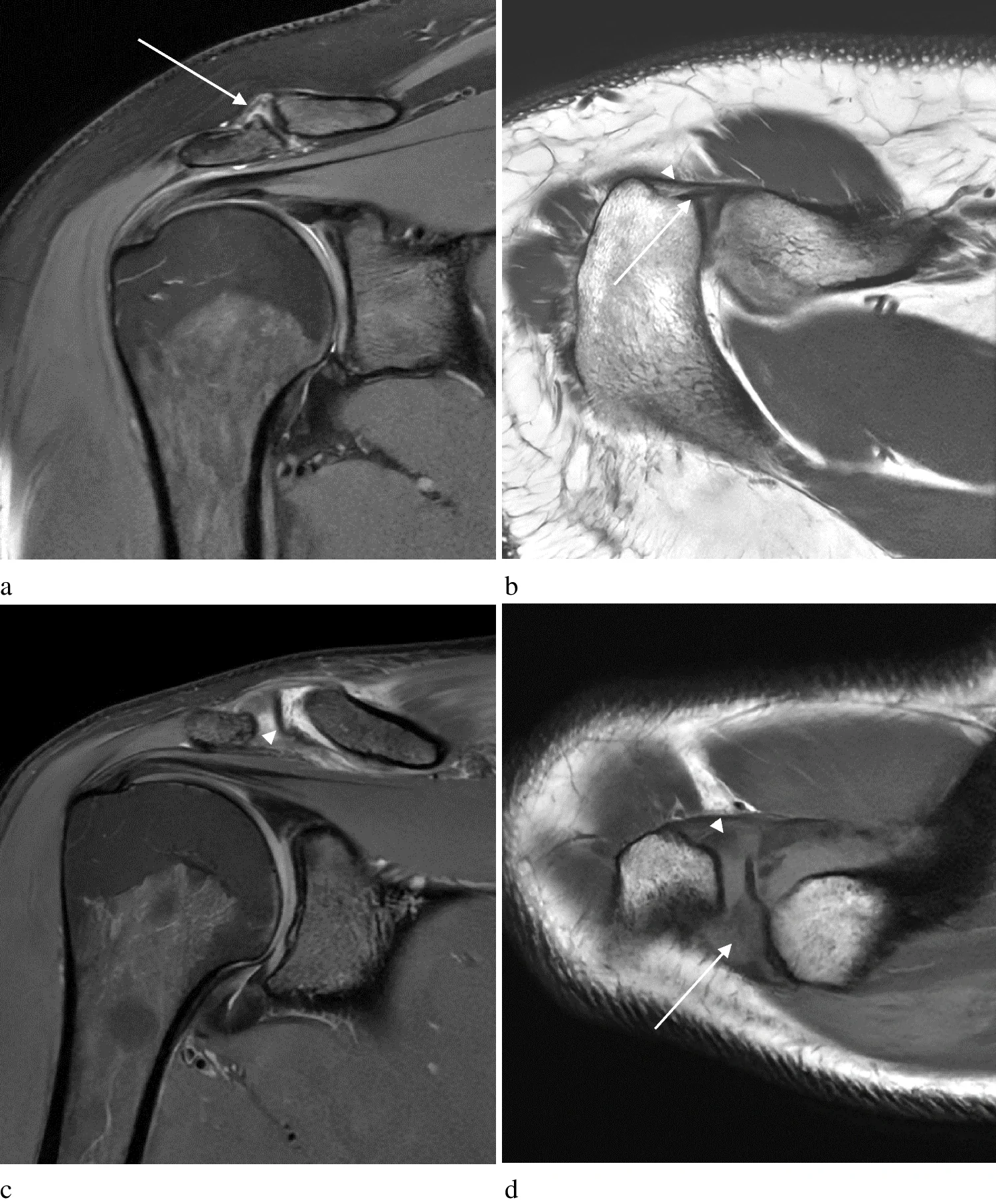
**a** A 21-year-old man with right shoulder pain following a hockey injury. MR image of the right shoulder showing a sprained acromioclavicular ligament in a coronal PD-weighted sequence. Edema is visible in the superior acromioclavicular ligament bundle (arrow). **b** The same injury on PD-weighted sequence parallel to the acromioclavicular joint, demonstrating a thickened, but not torn, anterior acromioclavicular ligament bundle (arrow) with an intact articular capsule (arrowhead), classified as Rockwood type I. **c** A 35-year-old man with right shoulder pain following a bicycle fall. MR image of the right shoulder showing a completely torn acromioclavicular ligament in a coronal PD-weighted image, with complete articular incongruency and fluid delineating the vertically oriented intra-articular fibrocartilaginous disc (arrowhead). **d** The same injury on a PD-weighted sequence parallel to the acromioclavicular joint showing complete disruption of the anterior (arrowhead) and posterior (arrow) acromioclavicular ligament bundles, classified as Rockwood type II

Clinical characteristics of patients are summarized in Table 1. The mean patient age was relatively young (38.4 ± 13.6 years). Of the 85 patients, 12 (14.1%) were female and 73 (85.9%) were male. Lesions were right-sided in 51 patients (60%) and left-sided in 34 patients (40%). Due to relatively easy access to MRI, the time interval between trauma and imaging was short (6.6 ± 4.5 days). A total of 41 (48%) examinations were performed on a 1.5 T MRI scanner with the remainder conducted on a 3 T MRI scanner. Examples of lesions are shown in Fig. 4 and Fig 5.

**Table 1:** Patients characteristics, time between trauma and MRI, and magnetic field strength

Patients (*n*)	Total = 85
Age (years)	38.4 ± 13.6
Female; male (*n*, %)	12 (14.1%); 73 (85.9%)
Right-sided lesion; left-sided lesion (*n*, %)	51 (60%); 34 (40%)
Time between trauma and MRI (days)	6.6 ± 4.5
Magnetic field (*n*, %)	
1.5 Tesla	41 (48%)
3 Tesla	44 (52%)

The intrareader reliabilities for each reader and each lesion are presented in Table 2. Intrareader reliabilities across sequences were very good for the selected combination of lesions of interest (i.e., ACL general condition and CCL general condition), with ICCs ranging from 0.81 (good; 95% CI = [0.76; 0.86]) to 0.90 (excellent; 95% CI = [0.87; 0.93]). No evidence of a difference was found in intrareader ICC values between pairs of readers for our condition of interest (all *p*-values below 0.009, see in Supplementary Material 2), indicating, as expected, that readers with different levels of experience showed comparable reliability across both 4- and 6-sequences protocols (Supplementary Material 2).

**Table 2:** Intrareader agreement for ACJ ligaments and muscle insertions by reader, comparing the standard protocol (4 sequences) with the standard protocol + additional ACJ acquisitions (6 sequences in total)

Parameters, ICC [95% CI]	Reader 1	Reader 2	Reader 3	Reader 4
ACL general condition	0.89 [0.84; 0.93]	0.80 [0.71; 0.87]	0.92 [0.88; 0.95]	0.76 [0.65; 0.84]
Superior portion	0.91 [0.87; 0.94]	0.65 [0.50; 0.76]	0.93 [0.90; 0.96]	0.77 [0.67; 0.85]
Inferior portion	0.96 [0.94; 0.98]	0.70 [0.57; 0.80]	0.96 [0.94; 0.97]	0.76 [0.65; 0.83]
Anterior portion	0.91 [0.86; 0.94]	0.67 [0.54; 0.78]	0.91 [0.87; 0.94]	0.81 [0.73; 0.88]
Posterior portion	0.89 [0.84; 0.93]	0.70 [0.57; 0.79]	0.93 [0.89; 0.95]	0.77 [0.66; 0.84]
CCL general condition	0.89 [0.83; 0.93]	0.83 [0.75; 0.89]	0.89 [0.83; 0.93]	0.90 [0.85; 0.93]
Trapezoid portion	0.92 [0.89; 0.95]	0.81 [0.72; 0.87]	0.88 [0.82; 0.92]	0.87 [0.81; 0.91]
Conoid portion	0.88 [0.82; 0.92]	0.81 [0.72; 0.87]	0.87 [0.81; 0.92]	0.88 [0.82; 0.92]
Deltoid muscle*				
Clavicular insertion	0.75 [0.64; 0.83]	0.64 [0.50; 0.75]	0.76 [0.65; 0.84]	0.81 [0.73; 0.88]
Trapezius muscle				
Acromial insertion	0.77 [0.67; 0.85]	0.33 [0.13; 0.51]	0.80 [0.70; 0.86]	0.75 [0.62; 0.84]
Clavicular insertion	0.68 [0.52; 0.79]	0.18 [−0.03; 0.38]	0.80 [0.71; 0.87]	0.59 [0.40; 0.72]
**Overall**	0.91 [0.89; 0.92]	0.74 [0.71; 0.77]	0.91 [0.90; 0.92]	0.84 [0.82; 0.86]
**All ACL and CCL**	0.91 [0.89; 0.92]	0.75 [0.72; 0.79]	0.91 [0.90; 0.92]	0.84 [0.81; 0.86]
**ACL and CCL general condition**	0.89 [0.86; 0.92]	0.81 [0.76; 0.86]	0.90 [0.87; 0.93]	0.86 [0.81; 0.89]

ACJ acromioclavicular joint, ACL acromioclavicular ligament, CCL coracoclavicular ligament

*The deltoid acromial insertion lesions were excluded from the table because of aberant numbers due to statistical limitations

The interreader agreements for each lesion and both sets of sequences are reported in Table 3. For the condition of interest, interreader agreements were excellent for both protocols (four sequences: ICC [95% CI] = 0.92 [0.90; 0.94]; six sequences: ICC [95% CI] = 0.93 [0.92; 0.95]), with no significant difference observed (ΔICC = –0.01, z = –1.03, p = 0.305). Furthermore, a significant equivalence of interreader agreement across all lesions was demonstrated between the 4- and 6-sequences protocols (*t*(12) = 2.946, *p* = 0.006). The detailed number of lesions (partial or complete tear) identified by the readers for each ligament, along with the percentage of findings that were concordant with the internal comparator, which, in the absence of surgical correlation, was defined as the results of the senior radiologist (reader 1) using all imaging sequences can be found in Supplementary Material 3.

**Table 3:** Interreader agreements based on standard shoulder protocol (4 sequences) and standard protocol + additional ACJ acquisitions (6 sequences)

Parameters, ICC [95% CI]	4 sequences	6 sequences	Difference	*z*-score	*p*-value
ACL general condition	0.92 [0.88; 0.94]	0.92 [0.88; 0.94]	diff = 0	*z* = −0.04	*p* = 0.968
Superior portion	0.89 [0.85; 0.93]	0.90 [0.86; 0.93]	diff = −0.01	*z* = −0.38	*p* = 0.703
Inferior portion	0.91 [0.87; 0.94]	0.90 [0.86; 0.93]	diff = 0.01	*z* = 0.26	*p* = 0.794
Anterior portion	0.88 [0.84; 0.92]	0.90 [0.86; 0.93]	diff = −0.01	*z* = −0.62	*p* = 0.532
Posterior portion	0.92 [0.89; 0.95]	0.92 [0.89; 0.95]	diff = 0	*z* = −0.09	*p* = 0.925
CCL general condition	0.93 [0.90; 0.95]	0.94 [0.92; 0.96]	diff = −0.02	*z* = −1.21	*p* = 0.227
Trapezoid portion	0.95 [0.94; 0.97]	0.95 [0.92; 0.96]	diff = 0.01	*z* = 0.82	*p* = 0.413
Conoid portion	0.94 [0.91; 0.96]	0.95 [0.93; 0.97]	diff = −0.02	*z* = −1.23	*p* = 0.22
Deltoid muscle					
Acromial insertion	0.29 [0.01; 0.51]	0.16 [−0.18; 0.42]	diff = 0.13	*z* = 0.9	*p* = 0.367
Clavicular insertion	0.89 [0.85; 0.93]	0.93 [0.90; 0.95]	diff = −0.03	*z* = −1.59	*p* = 0.112
Trapezius muscle					
Acromial insertion	0.84 [0.77; 0.89]	0.87 [0.82; 0.91]	diff = −0.03	*z* = −0.95	*p* = 0.34
Clavicular insertion	0.80 [0.72; 0.86]	0.83 [0.77; 0.89]	diff = −0.04	*z* = −0.83	*p* = 0.408
**Overall**	0.93 [0.92; 0.94]	0.94 [0.93; 0.94]	diff = −0.01	*z* = −2.16	*p* = 0.031
**All ACL and CCL**	0.92 [0.91; 0.93]	0.92 [0.91; 0.93]	diff = −0.01	*z* = −0.95	*p* = 0.344
**ACL and CCL general condition**	0.92 [0.90; 0.94]	0.93 [0.92; 0.95]	diff = −0.01	*z* = −1.03	*p* = 0.305

Based on records made by the senior musculoskeletal radiologist (reader 1), ACJ dislocations were reported in 75 patients (88.2% of the cohort) and Table 4 shows associated shoulder lesions in patients with and without ACJ ligamentous complex injuries. The most frequently observed associated lesion in acromioclavicular dislocation apart from muscular involvement was a distal clavicle anomaly (edema or fracture) found in 19 patients (25%). Supraspinatus tendon lesions rose to 17% (*n* = 13), of which 46% of them (*n *= 6) had a degenerative appearance.

**Table 4:** Intrareader omparison of associated shoulder lesions in patients with and without acromioclavicular ligament injuries

Associated lesions (*n*, %)	Patients without ACL or CCL lesion (*n* = 10)	Patients with ACL and/or CCL lesion (*n* = 75)
CAL	0 (0%)	3 (4%)
Deltoid muscle (edema or detachment)		
Acromial insertion	0 (0%)	2 (2.7%)
Clavicular insertion	0 (0%)	60 (80%)
Trapezius muscle (edema or detachment)		
Acromial insertion	0 (0%)	61 (81.3%)
Clavicular insertion	0 (0%)	63 (84%)
Humerus (bone marrow edema or fracture)	6 (60%)	1 (1.3%)
Glenoid part of the scapula (bone marrow edema or fracture)	2 (20%)	0 (0%)
Scapula (bone marrow edema or fracture)	0 (0%)	1 (1.3%)
Acromion (bone marrow edema, fracture)	1 (10%)	2 (2.7%)
Distal clavicle (bone marrow edema or fracture)	2 (20%)	19 (25.3%)
Sign of subacromial-subdeltoid bursitis	5 (50%)	4 (5.3%)
Supraspinatus tendon lesion (partial or complete)	3 (30%)	13 (17.3%)
Infraspinatus tendon lesion (partial or complete)	5 (50%)	2 (2.7%)
Teres minor tendon lesion (partial or complete)	1 (10%)	1 (1.3%)
Subscapularis tendon lesion (partial or complete)	6 (60%)	2 (2.7%)
Supraspinatus fatty degeneration according to Goutallier	0 (0%)	0 (0%)
Infraspinatus fatty degeneration according to Goutallier	0 (0%)	0 (0%)
Teres minor fatty degeneration according to Goutallier	0 (0%)	0 (0%)
Subscapularis fatty degeneration according to Goutallier	0 (0%)	0 (0%)
Long head of biceps tendon lesion (partial or complete)	2 (20%)	4 (5.3%)
Long head of biceps tendon sub/luxation	3 (30%)	1 (1.3%)
Labral tear	5 (50%)	4 (5.3%)
Chondral lesion	0 (0%)	0 (0%)
**Total**	10/85 (11.7%)	75/85 (88.2%)

## Discussion

The present study demonstrates that the use of dedicated ACJ ligaments MRI sequences does not provide evidence of a difference in the evaluation of the ACJ ligamentous complex compared to the standard shoulder MRI protocol in the context of an acute injury. This finding remains consistent regardless of the radiologist’s level of experience (junior vs. senior) or professional field of expertise (radiologist or orthopedic surgeon).

Acromioclavicular dislocation is a common injury, particularly among young males under the age of 35 [17–21], as reflected in our study population. Precise evaluation of the acromioclavicular ligaments is crucial, as their integrity directly influences the indication for surgical intervention. Intact or partially injured CCL (Rockwood types I and II) is managed conservatively [22], whereas complete CCL tears (Rockwood types IV, V, and VI) require surgical repair[23]. Management of Rockwood type III injuries, accounting for approximately 40% of all ACJ injuries, remains controversial [24] and is poorly characterized by radiography alone [25–27]. This underscores the limitations of the Rookwood classification, introduced in 1984, still considered as the reference standard, which relies exclusively on radiographic findings. Consequently, MRI has gained importance in both clinical grading and therapeutic decision-making [3–5, 28, 29]. Patients with high-grade injuries may benefit from the improved vision of anatomical details provided by MRI, especially when distinguishing between arthroscopic and open surgical approaches [29]. Therefore, MRI should be systematically performed whenever clinical suspicion of acromioclavicular instability exists.

To our knowledge, no previous study has compared the diagnostic value of shoulder MRI with and without ACJ sequences for the assessment of the ligaments. Moreover, no prior research has attempted to evaluate individually the four distinct anatomical portions of the ACL using a sequence parallel to the ACJ joint. Owing to the high spatial resolution of dedicated ACJ sequences with a slice thickness of 2 mm, this study allows detailed evaluation of the ACL sub-portions as well as the conoid and trapezoid components of the CCL. The coronal oblique plane, as previously described in the literature, enables full visualization of the CCL in a single image [5]. However, the findings indicate that this does not show signs of improvement in the analysis of the various CCL injuries. Intrareader reliability for CCL assessment remains good to excellent across all readers, corroborating that the assessment in the standard coronal plane is highly sufficient in the evaluation of this ligament regardless of the reader’s level of expertise.

In case of ACJ dislocation, treatment decisions are primarily driven by cranio-caudal instability as visualized on standard radiographs. However, anteroposterior instability can also lead to chronic pain and is not always apparent on x-rays. This underlines the importance of evaluating the anterior and posterior portions of the ACL on MRI. The additional sequence parallel to the ACJ joint, as described in this study, has never been reported in the literature before. Despite this novelty, it did not provide a statistically significant advantage in characterizing anterior and posterior bundles compared to the standard axial shoulder plane. This could be attributed to the inherent difficulty in assessing these structures, which are sometimes not identifiable on imaging. It is also reasonable to hypothesize that isolated damage to a single portion of the ACL seems unlikely. In most cases, when the superior and inferior portions are involved, the anterior and posterior bundles are likely also affected. While a more detailed assessment of ACL substructures may offer additional insight to orthopedic surgeons, it currently does not alter surgical decision-making. Due to the challenge of distinguishing the ligaments from the joint capsule, the term “ligamentocapsular complex” is likely more appropriate.

The muscle insertions of deltoid and trapezius are frequently reported in the literature as associated injuries in cases of acromioclavicular dislocation [5], a finding that is consistent with the results of this study which showed a high rate of muscle involvement in the subgroup with ACJ injury. The relatively low intrareader reliability for assessing muscle insertions may be partly attributed to the inherent difficulty in distinguishing between muscle edema and fiber detachment, particularly in the presence of substantial surrounding edema. Importantly, such muscle injuries do not influence current surgical decision-making in clinical practice. Beyond these muscle-related findings, associated shoulder injuries in the setting of acromioclavicular dislocation were infrequent, occurring in less than 5% of cases. This low incidence can be partially explained by the relatively young age of the study population. These results suggest that routine glenohumeral arthroscopy in the context of ACJ dislocation may not always be justified and should be reconsidered.

Moreover, omitting additional sequences results in a 30% and 20% reduction in total scan time on 1.5 T and 3 T scanners, respectively, leading to a more efficient use of imaging resources and significant time savings. In addition, interpretation time for the radiologist is shorter. This approach also improves departmental workflow and ultimately reduces costs. In this context, the study contributes to a broader initiative aimed at reducing MRI protocols, thereby promoting a more responsible and efficient approach to radiology, with greater consideration for resource utilization and environmental impact [30–34].

This study has some limitations due to its retrospective and monocentric design, which introduces the potential for selection bias. The lack of surgical confirmation as a reference standard may limit definitive correlation with imaging findings. However, prior studies have demonstrated that MRI is reliable and accurate for evaluating both soft tissue and osseous injuries in the context of acromioclavicular dislocation [3–5, 29].

In conclusion, this retrospective cohort does not support the routine use of supplementary ACJ planes, as they do not show evidence of improvement in diagnostic performance, regardless of reader experience or professional field of expertise. Additional sequences may be reserved for select cases where enhanced visualization of specific ligamentous structures is clinically justified. 

## Supplementary Information

Below is the link to the electronic supplementary material.

Supplementary file1 (DOCX 31 kb)

Supplementary file2 (DOCX 31 kb)

## Abbreviations

ACJ: Acromioclavicular joint
ACL: Acromioclavicular ligament
CAL: Coracoacromial ligament
CCL: Coracoclavicular ligament
CI: Confidence interval
ICC: Intraclass correlation
MRI: Magnetic resonance imaging
PD: Proton density

## Declarations

**Ethical approval**

Institutional Review Board approval was obtained (CER-VD BASEC-ID 2024-00840) on May 28, 2024. The study was performed in accordance with the Declaration of Helsinki and with its later amendments.
