# Supplementary material for: Assessment of dedicated MRI sequences in the evaluation of the acromioclavicular joint dislocation—A retrospective study
Source: Skeletal Radiol. 2026 Mar 23;55(7):1605–15. doi: 10.1007/s00256-026-05176-5 (PMC13649895; doi:10.1007/s00256-026-05176-5)
Supplement: Supplementary file 1 [file 256_2026_5176_MOESM1_ESM.docx]

| Supp. Material 1. Example of imaging parameters (variations may occur) | | | | |
| --- | --- | --- | --- | --- |
|  | **1.5 T** | | **3 T** | |
| Parameters | **Coronal oblique** | **Parallel to ACJ** | **Coronal oblique** | **Parallel to ACJ** |
| TR (ms) | 5500 | 2800 | 2200 | 1600 |
| TE (ms) | 70 | 60 | 45 | 45 |
| FA (°) | 160 | 160 | 111 | 111 |
| FOV (mm) | 180 | 180 | 120 | 120 |
| Matrix (pixels) | 320 x 224 | 320 x 224 | 380 x 348 | 380 x 348 |
| Slice thickness (mm) | 2 | 2 | 2 | 2 |
| Gap (mm) | 2 | 2 | 2 | 2 |
| Slices (n) | 30 | 20 | 20 | 20 |
| AT (min.s) | 3.43 | 2.20 | 2.08 | 1.47 |
| TR time of repetition, TE echo time, FA flip angle, FOV field of view, AT acquisition time, ACJ acromioclavicular joint | | | | |
