# Supplementary material for: Assessment of dedicated MRI sequences in the evaluation of the acromioclavicular joint dislocation—A retrospective study
Source: Skeletal Radiol. 2026 Mar 23;55(7):1605–15. doi: 10.1007/s00256-026-05176-5 (PMC13649895; doi:10.1007/s00256-026-05176-5)
Supplement: Supplementary file 2 [file 256_2026_5176_MOESM2_ESM.docx]

| Supp. Material 2. Comparison of intra-reader ICC values between reader pairs for all lesions and for ACL general condition & CCL general condition. | | | | | | |
| --- | --- | --- | --- | --- | --- | --- |
|  | | | | | | |
| Pairing (difference, z-score, *p*-value) | Overall |  |  | ACL & CCL general condition | | |
| Reader 1 vs reader 2 | diff = 0.18 | z = 2.21 | *p* = 0.027 | diff = 0.08 | z = 1.88 | *p* = 0.06 |
| Reader 1 vs reader 3 | diff = 0.02 | z = 0.25 | *p* = 0.81 | diff = 0.01 | z = 0.32 | *p* = 0.75 |
| Reader 1 vs reader 4 | diff = -0.07 | z = -1.01 | *p* = 0.31 | diff = -0.03 | z = -0.93 | *p* = 0.35 |
| Reader 2 vs reader 3 | diff = 0.19 | z = 2.46 | *p* = 0.014 | diff = 0.09 | z = 2.2 | *p* = 0.028 |
| Reader 2 vs reader 4 | diff = 0.11 | z = 1.2 | *p* = 0.23 | diff = 0.04 | z = 0.95 | *p* = 0.34 |
| Reader 3 vs reader 4 | diff = -0.09 | z = -1.26 | *p* = 0.21 | diff = -0.04 | z = -1.25 | *p* = 0.21 |

*ICC intraclass correlation coefficient, ACL acromioclavicular ligament, CCL coracoclavicular ligament*
